# Effectiveness and Remediation Mechanisms of Geo-Electrochemical Technology for Arsenic Removal in Paddy Soil from Northern Guangxi

**DOI:** 10.3390/toxics13090728

**Published:** 2025-08-29

**Authors:** Yuxiong Jiang, Meilan Wen, Yao Sun, Panfeng Liu, Yunxue Ma, Caiyun Zhang, Xiaohan Zhang

**Affiliations:** 1 School of Earth Sciences, Guilin University of Technology, Guilin 541006, China; yuxiongjiang@glut.edu.cn (Y.J.); 1020220051@glut.edu.cn (Y.M.); m13595705541@163.com (C.Z.); 1020230054@glut.edu.cn (X.Z.); 2 Collaborative Innovation Center for Non-Ferrous Metal Mineral Exploration and Resource Efficient Utilization, Guilin University of Technology, Guilin 541006, China; 3 Guangxi Key Laboratory of Hidden Metal Mineral Exploration, Guilin University of Technology, Guilin 541006, China; 4 Shanxi Institute of Geological Survey Co., Ltd., Taiyuan 030006, China; 13068035453@163.com

**Keywords:** geo-electrochemical technology, arsenic pollutants, remediation effect, remediation mechanism, northern Guangxi

## Abstract

Arsenic pollution in paddy soil is a major environmental issue, and its remediation has become a subject of broad interest. Geo-electrochemical technology has been shown to have significant potential for remediating heavy metal-contaminated soils in recent years. Taking contaminated paddy soil from northern Guangxi as the research subject, this study aims to assess the effectiveness of geo-electrochemical technology for arsenic remediation. An orthogonal experimental design was used to identify the optimal combination of parameters, including power supply duration, voltage gradient, power supply mode, and electrolyte type. The arsenic removal efficiency was thoroughly assessed, and the underlying remediation mechanisms associated with geo-electrochemical technology combined with EDTA-2Na were extensively investigated. The findings revealed a substantial decrease in the residual arsenic fraction after treatment, accompanied by a substantial increase in the mobility and bioavailability of arsenic. The maximum removal rate of arsenic from the soil was determined to be 19.59%. Among the analyzed factors, electrolyte type exerted the most significant influence on the arsenic removal efficiency, followed by power supply duration and voltage gradient, while the impact of the power supply mode was less significant. The optimal remediation effect was achieved under the following conditions: a power supply duration of 108 h, a voltage gradient of 0.6 V/cm, continuous power supply mode, and the use of EDTA-2Na as the electrolyte. The multiple strong coordinating atoms in EDTA-2Na can form stable chelates with Fe^3+^ and Al^3+^ bound to arsenic in the soil, thereby causing the desorption of arsenic. The integration of geo-electrochemical technology with EDTA-2Na forms a synergistic multiphase electrochemical reaction mechanism, significantly improving the overall remediation efficiency in arsenic-contaminated soils.

## 1. Introduction

Soil is considered a vital part of the natural environment and a fundamental resource for the production of food, playing an essential role in sustaining the survival of human life and ecosystems [1]. However, the rapid expansion of agriculture and modern industries has intensified heavy metal contamination in soils, resulting in serious ecological degradation and critical risks to environmental safety [2]. Heavy metals are known for their accumulation, persistence, and strong biological toxicity. Once released into the soil, they pose serious threats to the survival and development of humans and other living organisms [3,4]. Arsenic—a toxic metalloid and common soil pollutant—exerts both acute and chronic toxic effects and has been classified as a Group 1 carcinogen by the International Agency for Research on Cancer [5]. Arsenic exists in both inorganic and organic forms in soil, with its speciation significantly increasing its bioavailability and toxicity; in particular, inorganic arsenic tends to be more harmful compared to its organic counterpart [6,7,8]. Arsenic can enter the human body through the food chain, leading to health issues such as pigmentation, chronic lung disease, cardiovascular disease, and neurological disorders [9]. Arsenic contamination has been identified as a critical environmental challenge in China, with recent geochemical assessments indicating that groundwater arsenic concentrations exceeding the WHO guideline limit of 10 μg/L affect an area of approximately 580,000 km^2^. Furthermore, the average arsenic concentration in soils across China has been reported at 11.2 mg/kg, significantly exceeding the global average of 7.2 mg/kg [10].

Growing concerns over soil arsenic contamination have driven researchers to explore various remediation approaches, including soil replacement, solidification, chemical leaching, and phytoremediation [11]. However, these techniques are hindered by prolonged implementation processes and high operational costs, significantly limiting their large-scale application. In contrast to conventional remediation approaches, electrokinetic remediation has emerged as a promising technology due to its cost-effectiveness, operational efficiency, and environmental compatibility, earning recognition both domestically and internationally for its potential in soil remediation [12,13]. Electrokinetic remediation involves inserting electrodes into contaminated soil and applying a direct current to establish an electric field. This process promotes the directional migration of heavy metal ions or organic contaminants under the influence of the electric field, enabling their effective removal from the soil matrix [14]. Electromigration and electro-osmosis constitute the primary mechanisms of electrokinetic remediation. Electromigration involves the movement of charged ions in soil pore fluid toward oppositely charged electrodes under an electric field, while electro-osmosis refers to the directional flow of pore fluid induced by the applied electric field [15].

Geo-electrochemical technology allows for deep-penetrating geochemical explorations, employing physical principles to assess the geochemical composition of the Earth. The principle of this approach involves applying an external electric field to the surface soil, prompting the migration of charged substances associated with deep ore bodies within the near-surface loose layer and their accumulation on the element collector surrounding the electrode. Through analysis of the ore-forming elements accumulated on the element collector and identifying anomalies, blind ore deposits can be effectively detected [16,17,18,19]. The migration and enrichment of ore-forming elements from deep-seated blind ore bodies to the surface provides a new perspective regarding the application of geo-electrochemical technology to remediate heavy metal contamination in farmland soils.

In recent years, significant progress has been achieved in applying geo-electrochemical technology for the remediation of soil heavy metal contamination. Sun et al. [20] applied geo-electrochemical technology to remove cadmium (Cd) from paddy soil in Quanzhou County, Guilin City. They reported that geo-electrochemical treatment reduced the Cd concentration in paddy soil from 0.58 mg/kg to 0.39 mg/kg; meanwhile, Cd levels in rice grains remained below the national limit of 0.2 mg/kg, ensuring safe rice production. However, the reaction and migration mechanisms governing heavy metal removal from farmland soils using geo-electrochemical technology remain poorly understood. Moreover, research on arsenic remediation is limited, which has hindered the broader application of geo-electrochemical technology in the field of soil pollution and management.

This study addresses the urgent ecological and environmental challenge of remediating and restoring farmland soils contaminated with heavy metals. Taking arsenic-contaminated farmland soils in the northern Guangxi Zhuang Autonomous Region as the research subject, this study conducted orthogonal experiments using a geo-electrochemical system to systematically examine the effects of the power supply duration, voltage gradient, power supply mode, and electrolyte type. These investigations allowed for evaluation of the dynamic changes in soil pH and electrical current throughout the remediation process, the trends of arsenic speciation, and the overall effectiveness of the approach. The study clarified the mechanisms underlying the integrated use of geo-electrochemical technology and EDTA-2Na for remediating arsenic-contaminated soils, emphasizing the pathways of arsenic mobilization, migration, and transformation throughout the remediation process. This investigation provides valuable technical insights and scientific evidence to support the practical application of geo-electrochemical methods for remediation of As-contaminated farmland.

## 2. Materials and Methods

### 2.1. Soil Source

**Figure 1:**
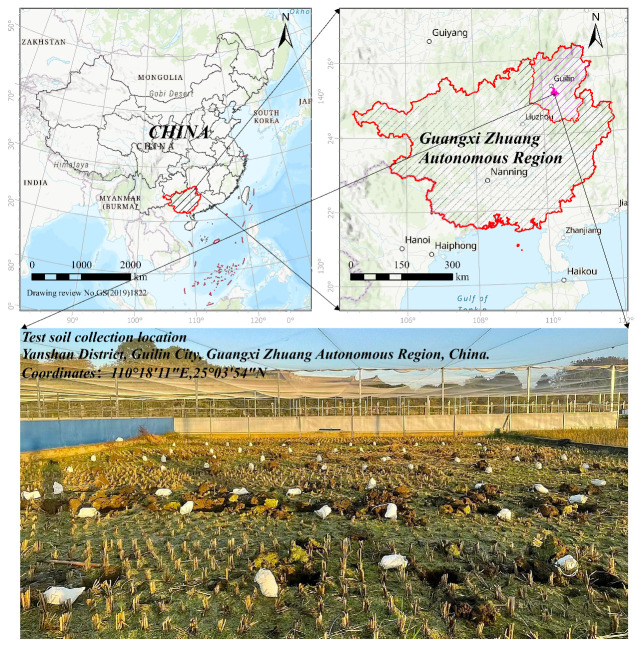
Test soil collection location.

The soil samples were collected from the experimental farmland of the Agricultural Science Institute located in the northern Guangxi Zhuang Autonomous Region, China. The specific sampling location is illustrated in Figure 1.

The experimental farmland site comprised continuous, evenly distributed rice paddies, where consistent soil fertility and arsenic contamination levels were ensured by applying uniform agricultural management practices throughout the area. The soil type is paddy soil; in particular, hydromorphic paddy soil derived from brown calcareous soil parent material. Field sampling was conducted during the fallow period of rice cultivation, with a sampling depth of 0–20 cm. The soil samples were divided into two portions: one was directly placed into the experimental device for the remediation experiment, while the other was stored in a pollution-free room to air-dry naturally. After air-drying, the soil samples were crushed with a wooden stick to remove plant debris and large particle impurities. The soil samples were then ground using an agate mortar and sieved through 10-, 20-, 100-, and 200-mesh sieves sequentially. The sieved samples were sealed and stored in a cool and dry place until subsequent determinations of pH, moisture content, and electrical conductivity, as well as arsenic content and speciation.

### 2.2. Experimental Setup

**Figure 2:**
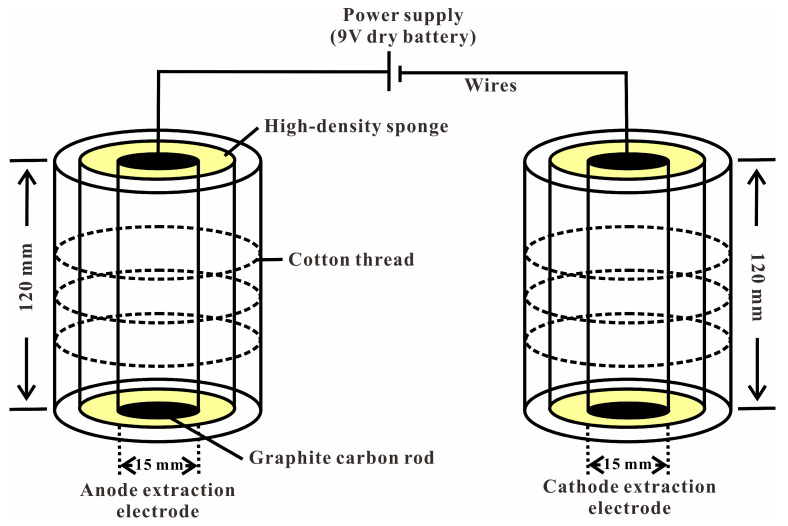
Schematic illustration of the geo-electrochemical system setup.

In this study, a low-voltage dipole geo-electrochemical extraction system, developed by the Institute for Prognosis of Concealed Ore Deposits at Guilin University of Technology [21], was employed as the core remediation apparatus. The structural configuration of the device is illustrated in Figure 2 [20]. The system primarily comprised anode and cathode extraction electrodes, along with a power supply and connecting wires. The extraction electrodes were constructed using cylindrical graphite carbon rods (height: 120 mm; base diameter: 15 mm) as the conductive core, wrapped in high-density sponges to enhance the efficiency of heavy metal adsorption.

**Figure 3:**
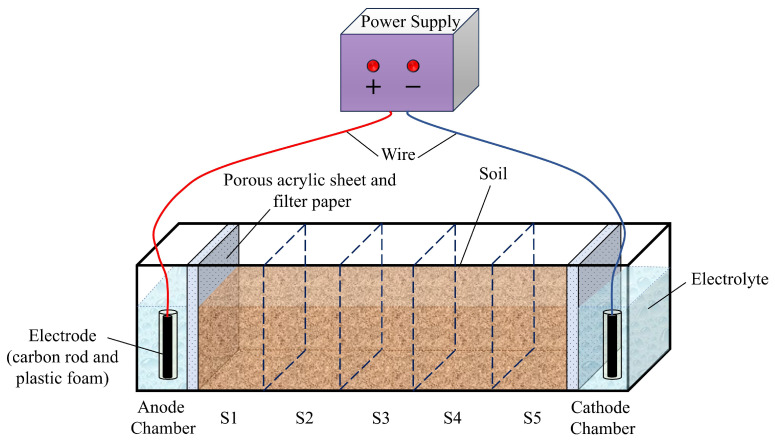
Schematic representation of the experimental setup used for geo-electrochemical remediation.

A specially designed experimental setup was constructed to meet the specific needs of the remediation study, as illustrated in Figure 3. The remediation device was constructed primarily from acrylic, featuring a tank with internal dimensions of 40 cm (length) × 10 cm (width) × 15 cm (height). It comprised three distinct compartments: an anode chamber, a cathode chamber (each with an internal length of 5 cm), and a soil chamber (with an internal length of 30 cm). To prevent soil particles from migrating into the electrolyte solutions within the electrode compartments, porous acrylic plates and multilayered filter papers of uniform composition were layered and placed at the junctions between the central soil chamber and the adjacent anode and cathode chambers. The electrodes were aligned in parallel within the anode and cathode chambers in order to maintain a consistent electric field distribution across the soil matrix. The electrodes were sequentially connected to a DC power supply and a multimeter via conductive leads. The soil chamber was divided equally into five sections based on proximity to the electrodes, designated as S1 (nearest the anode chamber), S2, S3, S4, and S5 (nearest the cathode chamber).

### 2.3. Multi-Factor Combinatorial Optimization

The remediation efficiency of geo-electrochemical technology is influenced by various interacting factors. Conducting a full factorial experiment would require extended durations, which may increase the risk of systematic errors due to time-dependent experimental drift. Therefore, the current remediation study was designed using an orthogonal experimental methodology to achieve time-efficient and cost-effective optimization. The use of an orthogonal experimental design enables the selection of representative test schemes from numerous combinations and identification of the optimal solution through outcome analysis. Moreover, it allows for the extraction of detailed information on individual factors through further data processing, thus providing more comprehensive experimental findings [22].

Based on a thorough review of previous electrokinetic remediation research, four major influencing factors were identified for this study, each of which was configured with three operational levels (as detailed in Table 1). The orthogonal experimental design was implemented using an L9 (3^4^) orthogonal array with the specific experimental conditions presented in Table 2. All nine experimental groups were conducted in three separate batches, with each group replicated three times under identical conditions to ensure the scientific reliability and accuracy of the data.

The energy consumption was calculated in this study using Equation (1):

(1) $$Ec=\frac{1}{m_{c}}\int UI\mathrm{dt}$$

where E_c_ denotes the specific energy consumption per unit mass of heavy metal contaminant removed from soil, with units of kW·h/mg; m_c_ represents the mass of heavy metal contaminants removed during the experiment, with units of mg; U indicates the voltage applied during the experiment, with units of V; I corresponds to the system’s current during the experiment, with units of A; and t signifies the experiment’s duration, with units of h.

### 2.4. Analytical Methods

The soil moisture content was determined following the procedure described in Soil and Agricultural Chemistry Analysis [23]. Electrical conductivity was measured using a DDS-307 conductivity meter (Kangyi, Shanghai, China) at a soil-to-water ratio of 1:5. Soil pH was assessed via the potentiometric method using a P20 pH meter (Youke, Shanghai, China) at a soil-to-water ratio of 2.5:1, while the pH of the electrolyte solution was directly measured. The total arsenic content in the soil was determined using an iCAP RQ ICP-MS, manufactured by Thermo Fisher Scientific, Waltham, MA, USA. The soil samples were digested sequentially using the four-acid digestion method (hydrochloric acid–nitric acid–perchloric acid–hydrofluoric acid). The speciation of arsenic in soil samples, both before and after remediation, was determined using the BCR sequential extraction procedure [24]. The analysis of total heavy metals in soil, along with the determination of total heavy metal concentrations in electrolytes, was performed by the Guilin Institute of Geology and Mineral Resources of China Nonferrous Metals. Soil sample digestion, heavy metal species extraction, and the analysis and processing of related samples were conducted at the Key Laboratory of Earth Sciences, Guilin University of Technology.

The sample testing process was subject to rigorous quality control measures, including the implementation of three sets of parallel samples for each group during sample analysis, ensuring a relative standard deviation of <10%. The limits of detection and reporting rates for total heavy metals and each form of determination were in accordance with the quality requirements for sample analysis.

### 2.5. Data Processing

This study employed IBM SPSS Statistics 26.0 for data analysis, while Origin 2020 and CorelDraw 2020 were used to generate graphical representations.

## 3. Results and Discussion

### 3.1. Physical, Chemical, and Contaminant Characteristics of Soil

The basic physicochemical properties and arsenic content of the tested soil are presented in Table 3. The results indicate that the soil is weakly acidic, with a pH higher than the background level typical of Guangxi soils, likely due to the long-term application of lime (CaCO_3_) under continuous cultivation to neutralize soil acidity. The soil demonstrated low electrical conductivity (10.60 μs/cm), indicating low concentrations of soluble salts and minimal ion presence. Meanwhile, the soil moisture content was relatively high, at 45%, suggesting favorable water retention conditions. The arsenic content in the soil measured 63.30 mg/kg, which was approximately 2.5 times higher than both the risk screening threshold and the regional background level for Guangxi soils [25], demonstrating a certain level of arsenic contamination in the tested soil.

### 3.2. Changes in pH

**Figure 4:**
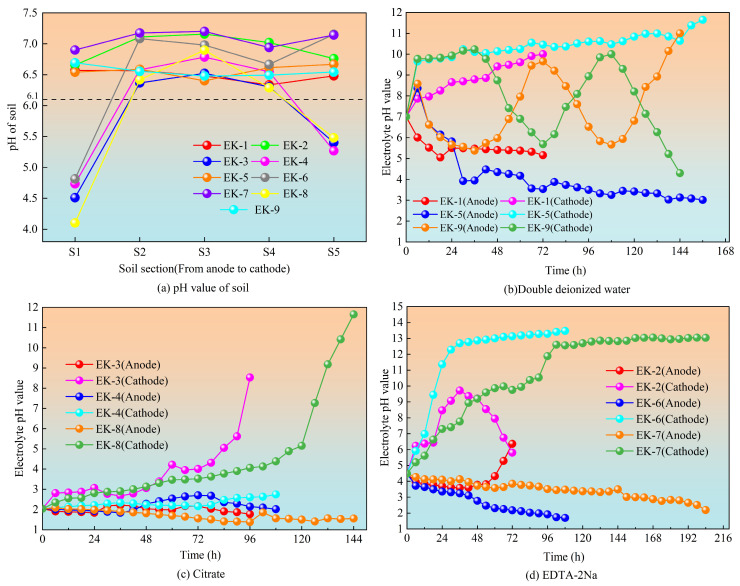
(**a**) Changes in soil pH; (**b**) pH variation with double deionized water as electrolyte; (**c**) pH variation with citric acid as electrolyte; (**d**) pH variation with EDTA-2Na as electrolyte.

Figure 4 presents the post-remediation pH values in different soil zones across the nine orthogonal experimental groups (Figure 4a), along with the temporal pH variations at the anode and cathode when using double deionized water (Figure 4b), citric acid (Figure 4c), and EDTA-2Na (Figure 4d) as electrolytes.

As shown in Figure 4a, post-remediation soils from the nine orthogonal experimental groups displayed pH values ranging from acidic to neutral (4.0–7.5) across all zones. Previous studies have suggested that wider soil acidification zones during geo-electrochemical remediation can effectively reduce the “focusing effect” and promote heavy metal desorption from soil particles, therefore enhancing the overall removal efficiency [26]. Overall, the soil pH across different zones (S1 to S5) in all experimental groups demonstrated an increasing trend, which can be attributed to the abundant H^+^ and OH^−^ ions generated via electrolysis. The H^+^ ions migrate toward the cathode, while OH^−^ ions move toward the anode through micro-electric field gradients and diffusion, resulting in a spatial pH gradient across the soil chamber. Thus, the soil pH progressively decreased in the vicinity of the anode and increased near the cathode.

As shown in Figure 4b, double deionized water was employed as the electrolyte in groups EK1, EK5, and EK9, enabling the pH variations in the anode and cathode chambers to directly reflect the intensity of water electrolysis. These three groups demonstrated a gradual decrease in anode electrolyte pH and a corresponding rise in cathode electrolyte pH after the initiation of geo-electrochemical remediation. The observed trend resulted from the electrolysis of water, generating H^+^ ions at the anode and OH^−^ ions at the cathode.

Figure 4c,d revealed that although the initial pH levels varied with variation in the electrolyte type, the overall pH variation patterns in the citric acid-based groups (EK3, EK4, EK8) and EDTA-2Na-based groups (EK2, EK6, EK7) were generally consistent with those observed in the deionized water groups. The EDTA-2Na groups demonstrated the greatest pH variation between the anode and cathode, followed by the citric acid and deionized water groups. This is primarily associated with the higher concentration of soluble ions in the EDTA-2Na and citric acid solutions, which enhanced the intensity of electrolytic reactions in both chambers and increased the variation in pH [27]. Periodic reversal of electrode polarity effectively reduced pH fluctuation amplitudes by alternating the roles of the electrodes, transforming the OH^−^-generating cathode into an H^+^-generating anode and vice versa, enabling H^+^ and OH^−^ neutralization to suppress extreme pH shifts.

The voltage gradient and power supply mode critically influenced electrolyte pH dynamics. Higher voltage gradients intensified electrolytic reactions, resulting in increased generation of H^+^ and OH^−^ ions and wider pH fluctuations. During later experimental stages, the continuous and intermittent power supply groups showed stabilized electrolyte pH levels, while electrode-reversal groups showed periodic pH oscillations due to alternating electrode polarities. This stabilization was attributed to the gradual depletion of electrolytes and a decline in water electrolysis activity, thus decreasing the generation of H^+^ and OH^−^ ions and shifting the system toward equilibrium [28].

Post-treatment, the soils predominantly remained alkaline. In such conditions, OH^−^ facilitates the desorption of arsenic oxyanions from soil surfaces and hinders their resorption, promoting the effective removal of arsenic [29].

### 3.3. Current Variation

The system current during geo-electrochemical remediation is intrinsically associated with the abundance of mobile ions within soil pores. The concentration of mobile ions directly influences the soil’s electrical conductivity, which in turn governs the magnitude of the electric current. The evolution of conductivity is driven by a dynamic balance between the generation and migration of mobile ions and their transformation into immobile forms, modulating current trends throughout the process [30,31].

**Figure 5:**
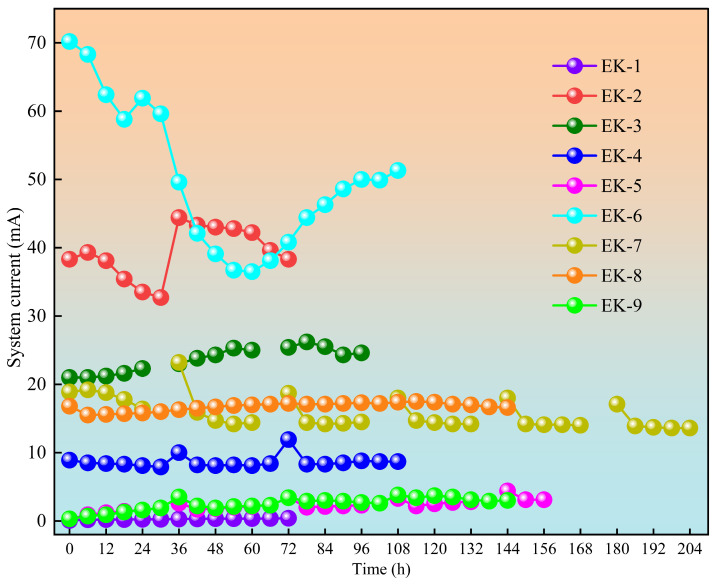
Current variation during geo-electrochemical remediation.

Figure 5 illustrates the variations in electric current throughout the electrokinetic remediation process. Upon initial energization, the experimental groups utilizing EDTA-2Na and citric acid demonstrated significantly higher current levels compared to the groups using double deionized water, reflecting the higher concentration of free ions present in the electrolyte solutions. At 6 h of operation, all experimental groups displayed an increasing current trend, indicating that ongoing electrolysis led to the migration of H^+^ ions into the soil, thus facilitating the desorption of additional ions. This process increased the concentration of mobile ions within the system, enhancing the soil’s electrical conductivity. As the experiment continued, the current levels remained relatively stable across the continuous power supply groups (EK1, EK8), intermittent power supply groups (EK3, EK5, and EK7), and electrode polarity reversal groups (EK4 and EK9). This stability persisted despite continuous H^+^ influx increasing ion concentration, as cationic species (e.g., Cu^2+^, Pb^2+^, Ca^2+^, Mg^2+^) migrating toward the cathode reacted with OH^−^ to form immobile heavy metal hydroxide precipitates, thus limiting further increases in conductivity [32]. When the two processes reach equilibrium, the influx of ions, their conversion into immobile forms, and the overall ion concentration stabilize, leading to a steady current magnitude.

The intermittent supply (EK3, EK5, EK7) and electrode-reversal groups (EK2, EK4, EK9) displayed characteristic current spikes upon power resumption, indicating transient ion mobilization as the electric field temporarily overcame soil resistance. The later decrease in current was primarily due to the gradual reduction in available mobile ions, with their continuous migration exceeding their regeneration within the system [33].

The voltage gradient critically regulated the current magnitude. In the citric acid groups, the current followed the order EK3 (0.9 V/cm) > EK8 (0.6 V/cm) > EK4 (0.3 V/cm)—a trend consistent with those observed in the EDTA-2Na and double deionized water groups. Higher gradients enhanced water electrolysis, producing more charge carriers and increasing the current. Under fixed voltage gradients, the current intensities consistently followed the order EDTA-2Na > citric acid > double deionized water across all power modes; this was primarily attributed to EDTA-2Na’s stronger chelating ability, allowing it to more effectively release soil-bound ions than citric acid [34].

### 3.4. Analysis of Arsenic Speciation in Post-Remediation Soil

Previous studies have indicated that arsenic in agricultural soils primarily exists in five speciation forms: Water-soluble fraction (T1), mild acid-soluble fraction (T2), reducible fraction (T3), oxidizable fraction (T4), and residual fraction (T5) [35]. The ease of removing these arsenic fractions using geo-electrochemical technology decreases in the following order: T5 > T4 > T3 > T2 > T1, indicating the increasing difficulty of extraction with each successive fraction [36].

**Figure 6:**
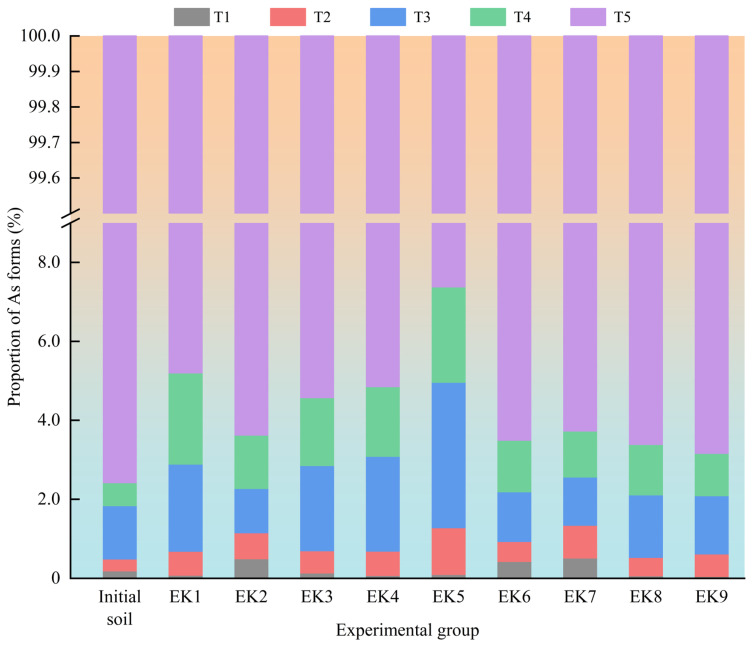
Distribution of arsenic speciation types in soil sampling zones at the end of the experimental test for each group.

Figure 6 illustrates the average proportions of these arsenic speciation types in post-remediation soils across the experimental groups. The residual fraction was identified as the predominant form of arsenic in the investigated soils, while the water-soluble fraction was the least abundant. After geo-electrochemical remediation, the residual fraction declined across all groups, accompanied by an increase in the more labile fraction. The observed findings confirm that geo-electrochemical technology promotes the transformation of stable residual arsenic into more mobile forms under enhanced conditions, facilitating its desorption and removal through the application of a micro-electric field, which is consistent with previous research findings [37]. The consistently high residual fraction is likely due to the presence of arsenic in stable mineral forms such as FeAsO_4_·2H_2_O [38]. FeAsO_4_·2H_2_O forms a dense crystalline structure through strong coordination bonds between Fe^3+^ and AsO_4_^3−^, resulting in extremely low solubility under ambient conditions (*Ksp* ≈ 10^−20^) and high resistance to natural weathering or chemical dissolution [39]. Studies have demonstrated that heavy metals bound to reducible Fe/Mn oxides readily dissolve under reducing conditions [40]. In groups EK2, EK6, and EK7, the use of the reductive electrolyte EDTA-2Na increased the desorption of reducible arsenic, leading to a decreased proportion of this fraction compared to pre-treatment levels.

In summary, geo-electrochemical technology enhances the mobilization of heavy metal species in soil by transforming insoluble fractions into water-soluble and more mobile forms, facilitating their effective removal. The micro-electric field drives the migration of contaminants from deeper soil layers to the surface, concentrating them near the electrodes to be captured by adsorption units. For effective practical application, periodic replacement of the electrolyte solution should be performed to remove dissolved metals, along with targeted excavation of soil around the electrodes to minimize secondary contamination risks due to the enrichment of surface metals. Furthermore, regular replacement of adsorption materials is essential to maintain the system’s effectiveness and ensure continuous arsenic removal from the soil.

### 3.5. Analysis of Arsenic Removal Results

**Figure 7:**
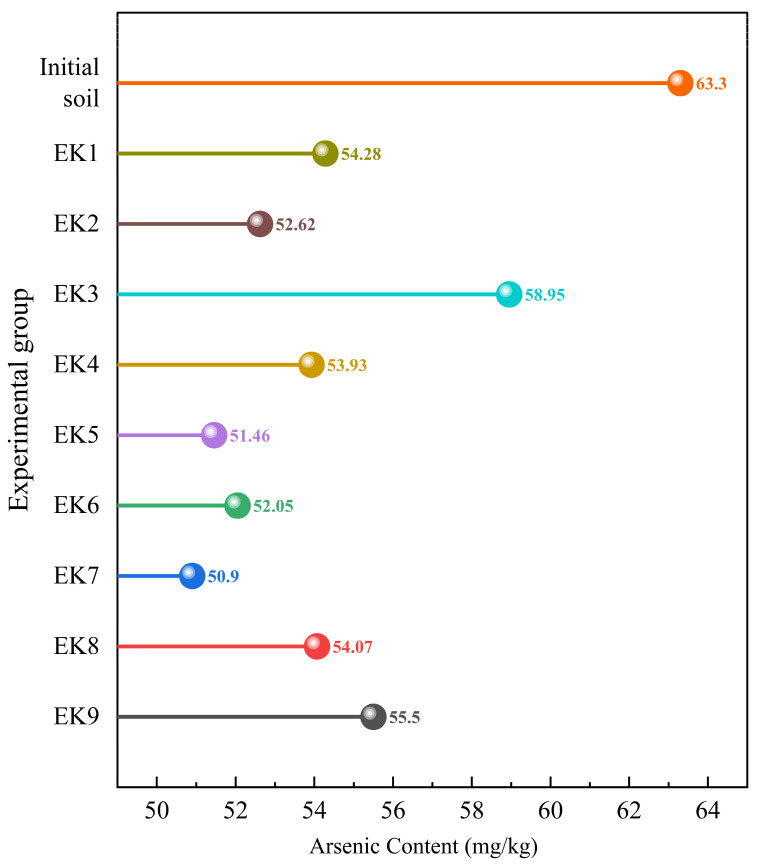
Comparison of arsenic content before and after geo-electrochemical remediation.

The results regarding the arsenic content, the orthogonal range analysis, and ANOVA for geo-electrochemical remediation of arsenic-contaminated soil are summarized in Figure 7, Table 4, and Table 5. As shown in Figure 7, the arsenic concentration in soil decreased significantly after the remediation process in all groups, with the EK7 group exhibiting the most pronounced removal efficiency. As shown in Table 4, the maximum arsenic removal rate reached 19.59%; furthermore, the removal efficiency exhibited a strong negative correlation with the percentage of arsenic in the residual fraction. Among the tested factors, the electrolyte type (*R* = 5.99) had the greatest impact on arsenic removal, followed by voltage gradient (*R* = 4.43) and power supply duration (*R* = 4.39), while the power supply mode exhibited minimal influence. The ANOVA results confirmed that electrolyte type, voltage gradient, and duration significantly influenced the arsenic removal rate (*p* < 0.05), where electrolyte type had the greatest effect (*SS* = 53.82), followed by voltage gradient (*SS* = 34.66) and duration (*SS* = 30.19). The optimal conditions identified were EDTA-2Na as the electrolyte, a voltage gradient of 0.6 V/cm, a duration of 108 h, and a continuous power supply.

Higher voltage gradients generate stronger electric fields, which intensify reactions within the remediation system and accelerate pH fluctuations. The generation of H^+^ ions resulting from these pH drops promotes the migration of metal ions, thus improving their removal efficiency. Furthermore, voltage gradients directly influence electro-osmotic flow—a key mechanism driving ion transport through soil pore water [41]. The remediation experiments revealed a paradoxical decline in arsenic removal efficiency when the voltage gradient exceeded 0.6 V/cm; this can likely be attributed to disruption of the soil pore structure and localized aggregation of colloidal particles caused by excessive voltage, thus hindering the transport of contaminants through soil pores. Prolonging the power supply duration improved the migration of arsenic by maintaining sustained micro-electric field exposure, underscoring the critical influence of operational time on the efficiency of heavy metal removal processes [42]. While extended operations enhance heavy metal extraction [43], excessively long durations increase energy consumption, raise costs, and can negatively impact the soil’s physicochemical properties. Continuous DC power maintains stable electro-osmotic flow, preventing ion re-adsorption during pauses. This directional transport drives arsenate anions (AsO_4_^3−^) toward the anode via electromigration, while As^3+^ cations migrate to the cathode through electro-osmosis.

EDTA-2Na exhibits a strong chelating ability with Fe^3+^ and Al^3+^, which adsorb the heavy metal element arsenic in soil. The following section of this article explores the underlying mechanism of the combined geo-electrochemical and EDTA-2Na approach enabling the effective remediation of arsenic-contaminated soil.

The specific remediation energy consumption for each experimental group is presented in Table 6. As shown in Table 6, EK1, EK5, and EK9 exhibited the lowest energy consumption. Considering an industrial electricity price of approximately CNY 0.6 per kW·h, the energy expenditure per unit arsenic removal was significantly lower in these groups compared to EK3 and EK6. This reduction is correlated with their use of deionized water as the electrolyte, with a lower ion concentration resulting in diminished system current. Despite favorable energy costs, these groups demonstrated lower arsenic removal rates, highlighting the need to balance cost-effectiveness with remediation efficiency. Regarding temporal efficiency, the 72 h treatment groups (EK1, EK2, EK3) showed limited remediation efficiency per unit time. Although EK7 (144 h treatment) displayed higher specific remediation energy consumption (1.41 kW·h/mg) than groups with shorter duration, its superior removal rate translated to enhanced effective remediation per unit time. This approach avoids the excessive energy surges and soil physicochemical degradation associated with prolonged operations, achieving an optimal balance between duration and energy expenditure. In summary, geo-electrochemical technology demonstrates promising application potential for contamination treatment; however, energy consumption remains a critical consideration. Strategic optimization of the voltage gradient, power supply mode, and electrolyte selection can simultaneously enhance the removal efficiency while reducing energy demand, thereby facilitating the practical implementation of this technology.

### 3.6. Mechanistic Analysis of Geo-Electrochemical Technology Coupled with EDTA-2Na for Remediation of Arsenic-Contaminated Soil

**Figure 8:**
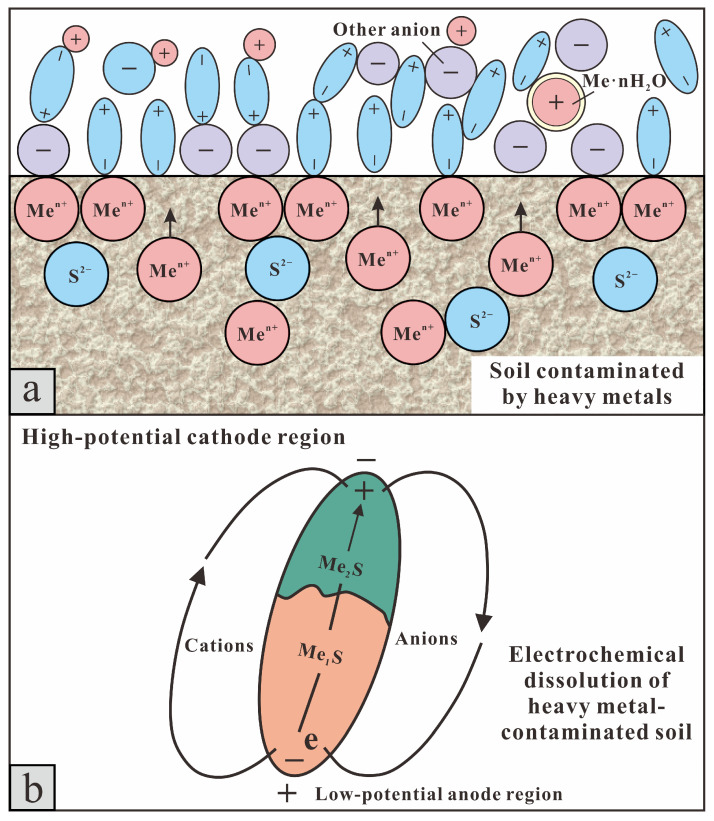
(**a**) Schematic diagram of the hydration reaction in heavy metal-contaminated soil; (**b**) Schematic diagram of the electrochemical dissolution process in heavy metal-contaminated soil.

In 1897, Helmholtz discovered that solid–liquid interfaces form electrical double layers [44]. In natural environments, soil particles containing heavy metals (solid phase) and surrounding fluids (groundwater or porewater; liquid phase) form numerous micro- to nano-scale solid–liquid interfaces. Differences in physicochemical properties between these phases induce charge separation, leading to the formation of electrical double-layer structures [45]. Under the influence of a micro-electric field, heavy metals are released from the solid phase, undergo hydration with polar water molecules at the liquid phase interface, and migrate as hydrated metal ions toward the electrodes for enrichment [46]. This is the electrochemical dissolution reaction occurring in heavy metal-contaminated soil. (Figure 8 and Chemical reaction Formula (2)):

(2) $$Me^{n+}+{n(H}_{2}{O)}^{-}\overset{\mathrm{Hydration}}{⇌}\mathrm{Me}\cdot nH_{2}O$$

where “Me” represents a certain soluble heavy metal element in the soil.

When external electric fields are applied, the soil electrochemistry is altered, disrupting inherent chemical barriers and releasing As-bearing charged colloids such as clay minerals and organic matter. These colloids then migrate continuously toward the electrode chambers through electromigration [47]. During electrokinetic extraction, micro-electric fields primarily drive the transport of arsenic through electromigration. Simultaneously, two electrochemical mechanisms occur within the soil layer: (1) electrostatic adsorption of oppositely charged particles at the electrodes and (2) electrolysis and complexation reactions at solid–liquid interfaces involving arsenic-laden colloids and soil solutions.

Therefore, arsenic remediation via geo-electrochemical treatment combined with EDTA-2Na involves two synergistic processes:

(i) Electrokinetic desorption and transport—the release of arsenic from soil matrices followed by mobilization driven by electromigration and electro-osmosis [9,42].
(ii) EDTA-2Na-assisted detachment and chelation—the displacement of arsenic species from soil surfaces through ligand exchange, followed by the formation of mobile As–EDTA complexes [48].

**Figure 9:**
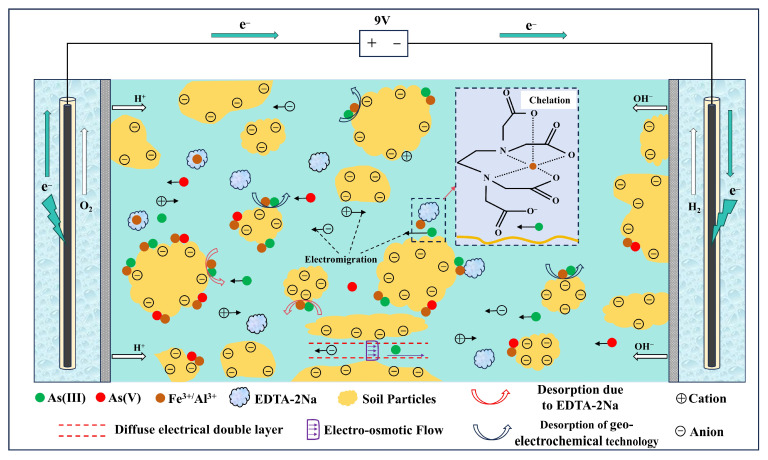
Arsenic remediation mechanism in soil using geo-electrochemical technology integrated with EDTA-2Na.

The mechanistic framework is illustrated in Figure 9.

In the soil environment, arsenic primarily exists as As(III) and As(V), mainly in the form of negatively charged arsenate oxyanions (e.g., HAsO_4_^2−^, H_2_AsO_4_^−^) [49]. EDTA-2Na, which contains multiple strongly coordinating nitrogen and carboxyl oxygen atoms, can form five- or six-membered chelate complexes with a small amount of As(III) [50]. Therefore, under specific conditions, arsenic transitions from stable residual fractions to mobile chelated forms, significantly increasing its mobility for subsequent electrokinetic removal [51]. EDTA-2Na also disrupts the adsorption equilibrium of arsenic on soil particles by chelating metal cations such as Fe^3+^ and Al^3+^, which are commonly bound to arsenic. This disrupts the adsorption equilibrium of arsenic on soil particle surfaces, causing its desorption into soil pore water [52].

The chelating action of EDTA-2Na converts arsenic into more mobile forms, while EDTA-2Na present in soil pores functions as a natural transport medium. The electromigration and electro-osmotic flow promoted by the geo-electrochemical system provide the driving force for the migration of mobile arsenic, synergistically improving the removal efficiency. Residual arsenic was found to be the predominant form in the soil, such that standalone geo-electrochemical remediation removed only a limited portion. Similarly, treatment with EDTA-2Na alone primarily facilitated the desorption or chelation of arsenic into the pore water without achieving effective extraction [53]. In the absence of EDTA-2Na, migrating arsenic may re-adsorb onto soil particle surfaces, leading to reduced remediation efficiency. The presence of EDTA-2Na reduces the likelihood of arsenic re-adsorption by forming stable complexes with Fe^3+^ and Al^3+^. Even if transient re-adsorption of arsenic onto soil particles occurs during electromigration, ubiquitous EDTA-2Na prompts its immediate re-desorption. This facilitates continuous arsenic transport under the applied electric field, ensuring highly efficient remediation [54,55].

In summary, the remediation effect of geo-electrochemical technology combined with EDTA-2Na in arsenic-contaminated soil is due to a multiphase electrochemical reaction mechanism, integrating the “effective collision-type” reactions driven by the micro-electric fields generated by the galvanic and electrolytic cells in the geo-electrochemical system with the “activated transition-type” chelation reactions of EDTA-2Na.

## 4. Conclusions

(1) Following geo-electrochemical remediation, the residual fraction of arsenic in the soil is transformed into more mobile forms, resulting in an increased proportion of mobile arsenic species.
(2) Group EK7 achieved the highest arsenic removal rate of approximately 19.59%. The results of the orthogonal experiment revealed that the type of electrolyte had the most significant impact on the arsenic removal efficiency, followed by the power supply duration and the voltage gradient, while the power supply mode had a minimal effect. The optimal conditions for removal of arsenic from soil were identified as a power supply duration of 108 h, a voltage gradient of 0.6 V/cm, continuous power supply mode, and EDTA-2Na as the electrolyte.
(3) The remediation effect of geo-electrochemical treatment combined with EDTA-2Na in arsenic-contaminated soil is due to a multiphase electrochemical reaction mechanism, integrating the “effective collision-type” reactions driven by micro-electric field effects derived from the electrokinetic process (involving galvanic and electrolytic cells) with the “activation transition-type” chelation reactions facilitated by EDTA-2Na.

## Figures and Tables

**Table 1:** Factors and corresponding levels.

Factor	Power Supply Duration	Voltage Gradient	Power Supply Mode	Electrolyte Type
Level 1	72 h	0.3 V/cm	Continuous	Double deionized water
Level 2	108 h	0.6 V/cm	Electrode reversal (every 36 h)	0.1 mol/L EDTA-2Na
Level 3	144 h	0.9 V/cm	Intermittent (24 h on/12 h off)	0.1 mol/L citric acid

**Table 2:** Orthogonal experimental design.

Experimental Group	Power Supply Duration (h)	Voltage Gradient (V/cm)	Power Supply Mode	Electrolyte Type
EK1	72	0.3	Continuous	Double deionized water
EK2	72	0.6	Electrode reversal	EDTA-2Na
EK3	72	0.9	Intermittent	Citric acid
EK4	108	0.3	Electrode reversal	Citric acid
EK5	108	0.6	Intermittent	Double deionized water
EK6	108	0.9	Continuous	EDTA-2Na
EK7	144	0.3	Intermittent	EDTA-2Na
EK8	144	0.6	Continuous	Citric acid
EK9	144	0.9	Electrode reversal	Double deionized water

**Table 3:** Statistical summary of basic physicochemical properties and arsenic content in soil.

Parameter	Result	Risk Screening Value	Risk Intervention Value	Background Value in Guangxi
pH	6.11			4.9
Electrical conductivity (μs/cm)	10.60			
Water content (%)	45			
The content of As (mg/kg)	63.30	30	150	24.80

**Table 4:** Orthogonal experimental results.

Run	Power Supply Duration (h)	Voltage Gradient (V/cm)	Power Supply Mode	Electrolyte Type	Arsenic Removal Rate (%)
EK1	72	0.3	Continuous	Double deionized water	14.24
EK2	72	0.6	Electrode reversal	EDTA-2Na	16.87
EK3	72	0.9	Intermittent	Citric acid	6.87
EK4	108	0.3	Electrode reversal	Citric acid	14.81
EK5	108	0.6	Intermittent	Double deionized water	18.70
EK6	108	0.9	Continuous	EDTA-2Na	17.77
EK7	144	0.3	Intermittent	EDTA-2Na	19.59
EK8	144	0.6	Continuous	Citric acid	14.57
EK9	144	0.9	Electrode reversal	Double deionized water	12.32
K_j1_	37.97	48.64	46.58	45.26	
K_j2_	51.27	50.14	43.99	54.22	
K_j3_	46.48	36.95	45.16	36.24	
k_j1_	12.66	16.21	15.53	15.09	
k_j2_	17.09	16.71	14.66	18.07	
k_j3_	15.49	12.32	15.05	12.08	
R	4.43	4.39	0.86	5.99	

**Table 5:** ANOVA results, * *p* < 0.05; ns, not significant.

Variance Source	Sum of Squares (*SS*)	Degrees of Freedom (*df*)	Mean Square (*MS*)	F-Value	Significance (*p* = 0.05)
Power supply duration	30.19	2	15.09	26.55	*
Voltage gradient	34.66	2	17.33	30.48	*
Power supply mode	1.14	2	0.57	1.00	ns
Electrolyte type	53.82	2	26.91	47.33	*
Total	119.94	8			

**Table 6:** Specific remediation energy consumption results.

Experimental Group	EK1	EK2	EK3	EK4	EK5	EK6	EK7	EK8	EK9
E_c_ (kW·h/mg)	0.02	2.39	3.52	0.9	0.16	4.34	1.41	2.35	0.41

## Data Availability

The data presented in this study are available on request from the corresponding author.
